# Ethical and Legal Considerations in Biometric Data Usage—Bulgarian Perspective

**DOI:** 10.3389/fpubh.2018.00025

**Published:** 2018-02-12

**Authors:** Jordan Deliversky, Mariela Deliverska

**Affiliations:** ^1^Department of National Security, University of Library Studies and Information Technologies, Sofia, Bulgaria; ^2^Department of Medical Ethics and Law, Faculty of Public Health, Medical University, Sofia, Bulgaria

**Keywords:** biometrics, data, human rights, protection, legislation

## Abstract

Ethical and legal considerations with regards to biometric data usage are directly related to the right to protection of personal data, which is part of the rights protected under the European Convention of human rights. Specific protection is required to the process and use of sensitive data which reveals certain personal characteristic and is related to the health status of individuals. Biometric data and information on individual upon which people could be identified based on specifics and distinguishing signs. Bulgaria, as a country progressing in terms of integration of digital technologies and as a European Union member state has adopted international and universal legal instruments related on the procession and use of digital data and data protection. On legislative and ethical grounds, it has been established the particular importance of not violating human rights and individual freedoms when processing and using personal data. It has been noted that the processing of special categories of personal data may be necessary for reasons of public interest in the field of public health and that is why under such circumstances it has been permitted the procession to be carried on without the consent of the data subject. Lack of transparency and lawfulness of the processing of personal data could lead to physical, tangible, or intangible damages where processing could lead to discrimination, identity theft, or identity fraud as a result of which may be significant adverse economic or social consequences. Increasingly, widespread use of biometrics in the implementation of medical activities requires the application of a new approach in terms of awareness regarding existing risks to the rights, ethics, and freedoms of all of us, as a user of medical service.

## Introduction

Human life has as its foundation the health of humans and that is the reason why health has to be effectively protected by solid actions all around the world. The prevention of health and the opportunity people to benefit from medical treatment has been recognized in legal acts as personal right. Acts recognizing such rights are the Charter of Fundamental Rights and the European Convention on Human Rights. The first act recognizes the right of access to preventive health care as well as the right to benefit from medical treatment. As part of the right to respect for private life, the European Convention on Human Rights proclaims the right to protection against collection and use of personal data.

In international legal act adopted in 1948, for the first time was recognized the right of privacy against interference from others, as in article 12 of the UN Universal Declaration of Human Rights, the right to privacy is proclaimed. The right to privacy as well as the right to health are both part of main fundamental human rights recognized in international legal instruments, such as the Universal Declaration of Human Rights, the United Nation International Covenant on Economic, Social and Cultural Rights of the United Nations, and the European Convention on Human Rights in Biomedicine.

In the treaty establishing the European Community and in numerous European Union legal, the protection of human health has been referred as an obligation, as the European union has responsibility for the health of third parties. The obligation for health protection is established under article 152 of the European Community Treaty, as in the European Community health policy, the improvement of personal health, the security, and the protection of human health has been a main focus.

An important fundamental human right with regards to biometric data usage is the right of protection with regards to the processing of personal data. Basic aspects of this right have been introduced in various international legal acts as the Charter of Fundamental Rights of the European Union (Article 8, paragraph 1), the Treaty on the Functioning of the European Union (Article 16 paragraph 1). According to legal instruments, the right to protection of personal data is a universal right, which is provided to everyone, as this protection has to comply with person’s fundamental rights and freedom.

The European Convention on Human Rights in its article 8 proclaims the right to personal data protection. Through this mechanism the right to respect private life has been guaranteed, as well as the right to home and correspondence. It lays down the conditions under which restrictions of the right are permitted (1).

At European Union Level, basic legal instrument related to protection of individuals with regards to the processing of personal data is Directive 95/46/EC. This legal act, also known as Data Protection Directive,^1^ refers to the free movement of personal data.

Reform of data protection issues at European Union level was put forward by the European Commission at the beginning of 2012 with regards to fit for the digital age, as the objective of all regulations in the area of personal data protection is to guarantee security. The security of the procession of personal data is an issue part of the Schengen Information System, a system supporting the law enforcement cooperation between Schengen States. Another objective which needs to be carefully observed is the determination of the conditions for date protection.

In times of digitalization, the strengthen of fundamental rights with regards to personal data is essential as this process would result to facilitation of management activities. Regulation is essential for simplifying rules for companies in the Digital Single Market internationally and on national level. In that aspect, Bulgaria does not make an exception.

Bulgaria, as a country progressing in terms of integration of digital technologies and as a European Union member state has adopted international and universal legal instruments related on the procession and use of digital data and data protection. The country has ratified the United Nations Universal Declaration of Human Rights and has incorporated the norms of the European Convention of Human Rights into its national legislative framework.

Bulgaria has transposed into national legislative act, norms of the European Union Directive 95/46/EC and as of January 1, 2002 the state enforces Personal Data Protection Act, promulgated in the State Gazette No. 1 of January 4, 2002 (2).

Bulgarian Personal Data Protection Act defines the term “personal data” which refers to information related to individuals. Personal data are information about individual who are identified or who can be identified by specific signs. This identification could be direct or indirect by ID or it can refer to one or more than one specific signs. The legal act also includes into its scope scientific approach on biometrics, as a science of identifying people. This identification distinguishes people according to their physical characteristics and it is performed by usage of various technologies analyzing characteristics as fingerprint, palm print, retina scan, voice patterns, facial structure, etc.

With regards to fundamental rights and freedoms, used data which is particularly sensitive by its nature, needs to be under special protection in terms of processing and usage (3). Data related to the health status of individuals as well as personal data revealing ethnic origin or racial origin falls into the scope of sensitive data. The legislation pays particular attention to specific categories of personal data and when it comes to processing of sensitive data such action could be established for health purposes when specific goals needs to be achieved for the benefit of the entire society or for the benefit of private individuals.

## Discussion

People unique and distinctive characteristics are those used by biometric technologies, when it is needed for identification of a person and that is the reason why these characteristics are being collected for automated verification of identity. Identification is not made by the system itself, as a biometric system compared to information submitted by individuals when a claim is made (4).

There are certain qualities of human characteristics which are mandatory as they shall be universal and persistent. Universal characteristics are the ones which shall be present with all human beings. An example of universal biometric characteristic is human fingerprint. When it comes to identification, there needs to consider the fact that persons may have lost a biometrically relevant characteristic. This could be resulted through accident, sickness, or peculiar circumstances. It is also important to be considered that in some ethnic groups of the population, some human characteristics are different or even less pronounced than average. This influences the way biometric systems work and that is the reason why general systems may be never accessible universally to all persons.

Fingerprints are one of the most distinguished and unique human biometric characteristic which contains ridges and valleys. Most biometric properties are based on patterns, and in fingerprints these patterns are formed by ridge-flows which are used by the classification systems for identification. Biometric systems use sensors to collect fingerprints, but sometimes fingerprints could be latent, as it is the case when a fingerprint is left by a person on an object. In cases when fingerprints are found and collected over the surface of on object, cooperation of the data subject is not required, and in such cases biometric data could be collected without the knowledge of the data subject.

There are various circumstances when procession of personal data could be performed without the knowledge of the data subject, and it is the case when processing may be necessary in the field of public health for reasons of public interest. It is particularly important not to be violated human rights and individual freedoms when personal data are processed, and that is why treatment of personal data should be subject to appropriate and concrete measures.

From legal perspective, the term “public health” should be interpreted within the meaning of Regulation (EC) 1338/2008 in the context of the treatment of special categories of personal data. All elements related to public and personal health, including morbidity and disability, which affect the need for health care and resources devoted to it, as well as providing health care and universal access, fall within the scope of this European Union legal act.

Data processing for the health of persons on grounds of public interest must not lead to the processing of personal data for other purposes by third parties, such as employers or insurance companies and banks (5).

The principle of transparency requires that any information about the data subject to be brief, clear, understandable, and easily accessible form, using clear and unambiguous formulations, including visualization. This information may be submitted in electronic form, such as through a website when it is addressed to the public. This is particularly important in cases where information platform is a technological complexity with a large number of participants, which actually hinders the data subject, as it prevents known and understood that gather related data, by whom and for what purpose.

Children are placed under special protection and are entitled with special protection when processing of information affects them. All information and communication regarding children should be provided with clear and plain language that can be easily understood.

Several principles are in line with legal regulation data processing on European Union level, as in the Directive on the protection of personal, the principles of honest, and transparent data processing has been established as well as the principle of limited conservation of data.

The data subject has to be informed of the existence of the processing operation and information needs to be provided on the scope of the procession. This information needs to be provided to the subject by the data controller as he has to ensure good faith and transparent handling of the data. Another important aspect that needs to be taken under consideration is the specific circumstances and context in which personal data is processed.

The principle of limited conservation of data refers to the form in which personal data is kept with regards to identification of data subjects. Data should be kept no longer than necessary for the purposes for which it has been collected. This rule also applies for the further procession of personal data.

Data should be anonymized, if the administrator wants to keep them once they have become obsolete and no longer serve their original purpose. Data are anonymous if all identifiable elements are removed from one set of personal data. Elements that could serve for re-identification of individuals should not be left in the data. When data are anonymized successful, there is no longer personal data.

The right to every person with regards to protection of personal data is established in Charter of Fundamental Rights, where in paragraph 2 of article 8 is established, that data must be processed fairly for specified purposes. The procession of personal data in relation to fundamental is required to be handled on the basis of the consent of the person concerned with regards to the procession of personal data (6).

Based on the provision of article 52, paragraph 1 of the Charter of Fundamental Rights, everyone has the right to access the data related to him and this norm also provides the right to the person to whom the data refers to have it rectified. Any restriction on this right must be provided by law. Such restriction should respect the essence of the relevant fundamental right and principle of proportionality.

The processing of personal data is regulated on national basis in Bulgarian legislative framework by the adoption of Personal Data Protection Act, where in 1, item 1 of the Supplementary Provisions of this act a definition of the term “personal data processing” has been implemented. The procession itself refers to actions which are performed upon personal data. These actions are performed by automated or other means, such as collection, recording, organization, storage, adaptation, or alteration, etc. The procession of personal data could be also performed by retrieval, consultation, use, disclosure by transmission, dissemination, making available, alignment or combination, blocking, erasure, or destruction.

There is a legal possibility in certain cases fingerprints to be categorized as “sensitive data” along with information about data which relate to the health, sex life, or human genome of a person. In this hypothesis, cases falling within the scope of Article 5, paragraph 1, item 3 of the Personal Data Protection Act needs to be aligned with the prohibition of processing of sensitive data.

The provision of the Bulgarian legal act establishing restriction of the processing of sensitive personal data does not apply in some cases, such as when the procession is required for the performance of specific rights and obligations of the controller. The restrictive provision is not applicable when the individual to whom sensitive data refers, has given explicit consent to the processing of his personal data, with the exception when a special law provides otherwise.

The restriction does not apply when processing is necessary for the protection of human life and health, but referring to the individual to whom the data relates. It is also not applicable in cases when the procession of personal data is carried out by non-profit organization of its legitimate activities with appropriate safeguards, provided that:

– the processing relates to the members of this profit organization or to persons who have regular contact with it;
– data are not disclosed to third parties without the consent of the individual to whom it relates.

When processing is carried out for the purposes of journalism, literary, or artistic expression, the restriction does not apply as long as the processing of personal data does not violate the right to privacy of the person to whom such data refers.

In cases when an individual makes public his own personal data or in cases when the processing is necessary for the establishment, exercise, or defense of legal claims, the restriction of the Bulgarian legal norm does not apply.

The restriction of the processing of sensitive personal data does not apply in cases when it is necessary for the purposes of preventive medicine or medical diagnosis, as well as in cases when providing or managing health services. The specific requirement in that case is that data is processed by a medical specialist obliged by law to observe professional secrecy.

In practical terms, a question arises, in which case it is permissible—the processing of personal data by scanning random fingerprint points. The answer to this question is directly linked to the provision of consent of the person whose personal data will be processed. To be able to do specific and informed statement, the person should be informed of the compulsory or voluntary nature of data provision and the consequences of refusing to provide them. In all cases, the person whose statement is required to provide information about the right of access and the right to correct the data, erasure or blocking of data collected, and the right to object to the processing of personal data in case of legal basis thereof.

Consent should be expressed in terms of the purpose of data processing, as after achieving the purpose of processing, personal data controller is obliged to destroy the information. Another possibility for the controller is to transfer the data to another administrator, but in that case he has to inform the Commission for Personal Data Protection in advance. The obligation for informing the Commission is set in situations when the transfer is provided by law and when there is an identity of purpose processing.

The term “individual’s consent” in Bulgarian Personal Data Protection Act has been defined expression of will. The consent has to be specific and it is obligatory to be freely given. By the expression of will, the individual to whom the sensitive personal data relates, agrees upon its procession. According to §1, item 13 of the additional provisions of the Personal Data Protection Act, consent should be always available in relation to the purpose of data processing.

Personal consent refers to the compulsory or voluntary nature of data provision and the consequences of refusing to provide consent, as in all cases people should be provided with information on the right of access and right of rectification, erasure or blocking of collected data. Information has to be also provided on the right to object to the processing of their personal data if there is legal basis for this.

Information processed for health purposes particularly with regards to management services and systems for health care and social care needs to be provided additional protection. Data procession for specific purposes may be related to treatment by management bodies and central national health authorities. Sensitive data may be processed for the purposes of quality control, information management, and overall monitoring of national and local levels of the health care system or social services.

When recording or disclosure of personal data is explicitly provided by law, or in cases when providing information is impossible or involves disproportionate efforts, it is not necessary to impose an obligation to provide information to the data subject. This would be the case in particular, where the processing is done for the purpose of archiving in the public interest, for the purposes of scientific or historical research or statistics. In this context, it should be taken into account the number of data subjects, timeliness of data, and appropriate safeguards established.

## Conclusion

Lack of transparency and lawfulness of the processing of personal data could lead to physical, tangible, or intangible damages, where processing could lead to discrimination, in identifying theft or identifying fraud as a result of which may be lead to significant adverse economic or social consequences.

Increasingly widespread use of biometrics in the implementation of medical activities requires the application of a new approach in terms of awareness regarding existing risks to the rights, ethics, and freedoms of all of us, as a user of medical service.

## Author Contributions

JD—ethical and practical aspects on the application of biometric data usage with regards to regulatory framework. Analysis of the identification of physical characteristics in technology usage with regards to personal biometric data. Specific cases on processing sensitive data with regards to the application of Personal Data Protection Act. Anonymization of personal biometric data with regards to administration of sensitive data. MD—international legal aspect of the consideration on usage of biometric data. The Charter of Fundamental rights with regards to the establishment of the right to every person to the protection of personal data. Public Health aspect with regards to personal data revealing.

## Conflict of Interest Statement

The authors declare that the research was conducted in the absence of any commercial or financial relationships that could be construed as a potential conflict of interest.

## Footnotes

^1^Data Protection Directive, OJ 1995 L 281, p. 31.

## References

[1] United Nations. Universal Declaration of Human Rights, Adopted by General Assembly Resolution 217 A(III) of 10 December. The UN Universal Declaration on Human Rights (1948). Available from: http://www.un-documents.net/a3r217a.htm

[2] Personal Data Protection Act. Prom. SG. 1/4 Jan. The Personal Data Protection Act (2002). Available from: http://ec.europa.eu/justice/data-protection/law/files/implementation/bg_data_protection_law_en.pdf

[3] Directive 2011/24/EU of the European Parliament and of the Council of 9 March 2011 on the Application of Patients’ Rights in Cross-Border Healthcare. The Directive 2011/24 EU (2011). p. 45–65. Available from: http://eur-lex.europa.eu/LexUriServ/LexUriServ.do?uri=OJ:L:2011:088:0045:0065:en:PDF

[4] White paper. Together for Health: A Strategic Approach for the EU 2008–2013. COM/2007/0630 Final. The White Paper (2007). Available from: https://ec.europa.eu/health/ph_overview/Documents/strategy_wp_en.pdf

[5] Regulation (EU) 2016/679 of the European Parliament and of the Council of 27 April 2016 on the Protection of Natural Persons with Regard to the Processing of Personal Data and on the Free Movement of Such Data, and Repealing Directive 95/46/EC (General Data Protection Regulation). The Regulation 2016/679. (2016). Available from: http://eur-lex.europa.eu/eli/reg/2016/679/oj

[6] Charter of the Fundamental Rights of the European Union. The Charter of the Fundamental Rights (2012). Available from: http://www.europarl.europa.eu/charter/pdf/text_en.pdf

